# Performance of Bayesian EWMA control chart with measurement error under ranked set sampling schemes with application in industrial engineering

**DOI:** 10.1038/s41598-023-40656-x

**Published:** 2023-08-28

**Authors:** Yuzhen Wang, Imad Khan, Muhammad Noor-ul-Amin, Amjad Javaid, Dost Muhammad Khan, Huda M. Alshanbari

**Affiliations:** 1 School of Science, Xi′an Technological University, Xi′an, 710032 China; 2 Department of Statistics, Abdul Wali Khan University Mardan, Mardan, Pakistan; 3 COMSATS University Islamabad, Lahore Campus, Lahore, Pakistan; 4 Pakistan Bureau of Statistics, Islamabad, Pakistan; 5 Department of Mathematical Sciences, College of Science, Princess Nourah bint Abdulrahman University, P.O. Box 84428, Riyadh, 11671 Saudi Arabia

**Keywords:** Engineering, Mathematics and computing

## Abstract

The objective of this study is to investigate the behavior of the Bayesian exponentially weighted moving average (EWMA) control chart in the presence of measurement error (ME). It explores the impact of different ranked set sampling designs and loss functions on the performance of the control chart when ME is present. The analysis incorporates a covariate model, multiple measurement methods, and a conjugate prior to account for ME. The performance evaluation of the proposed Bayesian EWMA control chart with ME includes metrics such as average run length and standard deviation of run lengths. The findings, obtained through Monte Carlo simulation and real data application, indicated that ME significantly affects the performance of the Bayesian EWMA control chart when RSS schemes are employed. Particularly noteworthy is the superior performance of the median RSS scheme compared to the other two schemes in the presence of ME.

## Introduction

Quality product is the ultimate satisfaction of the customer, which leads to an increase in the profitability of the producer. ISO-9000 certification not only leads the producers to produce high quality goods but also compels them to enhance the product quality to remain in the main steam of their business. Therefore, quality control techniques are the basic requirement to maintain quality standards during production and to save the raw material losses due to defective production. The statistical process control (SPC) provides techniques to perceive variations in the production processes prior to produce defective goods. Control charts (CCs) are valuable tools in SPC that enable quality engineers to efficiently monitor and enhance product quality. Shewhart^1^ was the first who floated the concept of the memory-less type of CCs only utilizing the current sample information. The CCs discussed in this context have a recognized capability to identify notable changes in the production process. The cumulative sum (CUSUM) and exponentially weighted moving average (EWMA) CCs, initially introduced by Page^2^ and Roberts^3^, are widely acknowledged as examples of memory-type CCs. This is because they make use of both current and historical sample data in their calculations. The CUSUM and EWMA CCs exhibit greater sensitivity in identifying small and moderate shifts in process parameters when compared to the classical Shewhart CC. These memory-type CCs, specifically EWMA and CUSUM, hold significant value in chemical and industrial production processes. Al-Omari and Bouza^4^ presents a literature review on RSS, a sampling method proposed as an efficient alternative to SRS for estimating population mean. The review covers different modifications of RSS and its wide range of applications. Ali et al.^5^ proposes a new non-parametric CC for monitoring manufacturing processes using sign statistics and RSS. Through simulation and comparison with existing CCs, it demonstrates robustness and improved detection of process shifts, validated by a practical application in substrate manufacturing. Abbas et al.^6^ suggested nonparametric CC for monitoring manufacturing processes, using the Wilcoxon signed rank test and RSS. The CC outperforms existing methods in detecting shifts in process location. Ali et al.^7^ studied nonparametric CC utilizing sign statistic and RSS for monitoring non-normal manufacturing processes. It effectively detects small and persistent shifts in process location, outperforming other alternatives, and is demonstrated using real-world data on piston ring diameter. This is primarily due to their enhanced ability to identify and promptly respond to minor to moderate disturbances, which often carry significant consequences. However, measurements of the variables required for construction of the CCs affect the efficiency of these charts during the process control, if not correctly measured.

The performance of the CCs is affected when measurement error (ME) creeps in during measurements of collected data regarding study variable(s) from sample chosen for construction of the CCs. Sometimes, the variation arises due to ME because of the deviation from actual measurement of the study variable which causes quality disturbances and undesired results. Consequently, the detection power of the CCs to find out-of-control signals is affected due to inaccurate measurements of the variables under study. To tackle the challenge of ME, researchers have introduced various methods for CCs. Mittag and Stemann^8^ investigates the impact of ME, specifically gauge imprecision, on the effectiveness of Shewhart-type $\overline{X} - S$ CCs. The study demonstrates that gauge imprecision can significantly affect the chart's ability to quickly detect process disturbances or result in incorrect signals of an out-of-control process state, depending on the timing of the error. This CC is based on the model $Y\, = \,X\, + \,\varepsilon$, where *X* embodies the true value of the study variable, $\varepsilon$ represents the random error term, and *Y* represents the observed value during the measurement process. Stemann and Weihs^9^ conducted a comprehensive study exploring the use of the EWMA $\overline{X} - S$ CC in the presence of ME. Linna and Woodall^10^ performed an analysis on the effectiveness of the $\overline{X} - S^{2}$ CC when ME is present. They employed a model $Y = A + BX + \varepsilon$, where A and B are known constants. In a related study, Linna et al.^11^ investigated the influence of ME on multivariate CCs. Maravelakis et al.^12^ suggested the application of the EWMA CC in the presence of ME, incorporating a model with linear covariates. Additionally, they investigated the implications of multiple measurements and a linearly increasing variance. Afterwards many authors studied the impact of ME on the efficiency of the CCs during industrial production e.g., Huwang and Hung^13^, Wu^14^, Hu et al.^15, 16^, Sabahno and Amiri ^17^, Riaz et al.^18^, Javaid et al.^19^, Noor-ul-Amin et al.^20, 21^ etc. Imran et al.^22^ examined the influence of ME on multivariate CUSUM CCs for compositional data (CoDa). ME had a significant impact on the CCs, particularly due to the error variance–covariance matrix. Subgroup size and the powering operator enhanced the CCs by reducing ARL. Conversely, an increase in the number of variables had an adverse effect, leading to higher ARL values. Real-world examples illustrated the practical application of these CCs in uncoated aspirin tablet manufacturing and muesli production machine monitoring. The researchers employed various techniques, including the covariate method, multiple measurements of the same sampled variable of interest, and the linearly increasing variance method. These techniques were used to compare the performance of CCs in the presence and absence of ME. The study’s conclusion emphasized that ME has a significant influence on the effectiveness of the CCs.

CCs typically rely on the classical approach, which considers only sample information and disregards prior knowledge. In contrast, the Bayesian analysis utilizes the posterior (*P*) distribution to seamlessly integrate sample information with prior knowledge. Menzefricke^23^ introduced a CC for the location parameter of a process by employing Bayesian theory. Within the Bayesian framework, the utilization of a LF serves the purpose of mitigating risks linked with the estimator, ultimately aiming to achieve the optimal Bayes estimator. In a study by Wu and Tian^24^, the CUSUM CC was recommended for monitoring both the process mean and variance, considering the weighted LF of the production process. Menzefricke^25^ conducted a study on variance CCs using the distribution applying Bayesian methodology. Additionally, Serel^26^ recommended a Bayesian-EWMA CC for both the process mean and variance, employing different LF techniques. Noor et al.^27^ investigates the application of Bayesian theory in control charting, incorporating symmetric and asymmetric LFs with informative and non-informative priors to create a hybrid EWMA (HEWMA) CC. The effectiveness of the offered chart is assessed using ARL and SERL measures, with simulations and a real data example provided. A novel adaptive EWMA (AEWMA) CC that integrates the Shewhart chart and EWMA chart to detect both large and small/moderate shifts in process mean suggested by Noor-ul-Amin and Noor^28^. The AEWMA chart is implemented using Bayesian theory, considering various LFs, informative priors, and performance measures like ARL and SDRL. A comparative study compares the suggested AEWMA chart with existing Bayesian EWMA charts, supported by a real-data example and a Monte Carlo simulation study for assessment. Noor et al.^29^ introduced a EWMA CC based on the Bayesian approach that incorporated diverse LFs, demonstrating the efficacy of their proposed approach.

All previous studies discussed in this article have predominantly focused on classical and Bayesian approaches utilizing SRS. In contrast, the objective of this research is to propose a Bayesian-EWMA CC that incorporates the concept of ME and employs various RSS techniques. We investigate two distinct LFs, namely the SELF and the LLF, to identify shifts in the location parameter of process. The effectiveness of the offered CC is assessed using the ARL and the SDRL. The structure of this paper is as follows: Sect. “Bayesian approach” discusses the Bayesian approach for the EWMA control chart and the employed LFs. Section “Ranked set sampling” provides an explanation of the different RSS designs. ME is explored in Sect. “Measurement error”, followed by the introduction of the recommended EWMA CC with ME using RSS applying Bayesian methodology in Sect. “Proposed Bayesian-EWMA CC based on with and without measurement error using various LFs under RSS schemes”. Section “Discussion on tables and main findings” presents the key findings, while Sect. “Real life data applications” demonstrates real-life data applications. Finally, Sect. “Conclusion” contains the conclusion of the article, and the limitations and recommendations are included in Sects. “Limitations of the study” and “Future recommendation”, respectively.

## Bayesian approach

The Bayesian approach is a powerful technique employed to estimate unknown population parameters by leveraging the *P* distribution, which incorporates information from both the sample data and prior knowledge. This methodology not only enables parameter estimation but also provides a robust framework for quantifying uncertainty and systematically updating beliefs. The prior distribution encapsulates our understanding or belief concerning an unknown population parameter prior to considering any specific evidence. It can be classified into two primary types: informative prior and non-informative prior. An informative prior is utilized when we have relevant information about the parameter of the prior distribution. On the other hand, the concept of a conjugate prior is explored, which occurs when the sampling distribution and prior distribution share the same family of distributions. The focus is on studying a variable *X* with a mean of $\theta$ and a variance of $\sigma^{2}$for the in-control process. To model the prior distribution, we choose a conjugate normal prior with parameters $\theta_{0}$ and $\sigma_{0}^{2}$, which can be mathematically represented as follows:

1 $$p\left( \theta \right) = \frac{1}{{\sqrt {2\pi \delta_{0}^{2} } }}\exp \left\{ { - \frac{1}{{2\delta_{0}^{2} }}\left( {\theta - \theta_{0} } \right)^{2} } \right\}$$

When there is insufficient information about a parameter in the prior distribution, it is known as a non-informative prior. In such cases, the prior has minimal impact on the *P* distribution. One common approach is to represent a non-informative prior as a uniform distribution. The probability density function (pdf) of a uniform distribution is typically expressed as $P\left( \theta \right)\, = \,c \, \surd \left( {n/\sigma^{2} } \right)$, where c represents a constant of proportionality.

The P distribution, characterized by parameter $\theta$, combines the prior distribution and sample distribution in the following manner:

2 $$p\left( {\theta |x} \right) = \frac{{p\left( {x|\theta } \right)p\left( \theta \right)}}{{\int {p\left( {x|\theta } \right)p\left( \theta \right)d\theta } }}$$

When we encounter a new data point *Y*, the PP distribution is derived by treating the *P* distribution as a prior distribution. This method enables us to integrate the information gleaned from the observed data and revise our predictions for the new data point. By employing the *P* distribution as a prior, we take into account the uncertainty associated with parameter estimation, resulting in a distribution that captures our refined understanding. Essentially, the PP distribution combines the existing data with the *P* distribution to furnish an informed prediction for the new data point *Y*. which is mathematized as

3 $$p\left( {y|x} \right) = \int {p\left( {y|\theta } \right)p\left( {\theta |x} \right)d\theta }$$

In the Bayesian theory framework, LFs play a crucial part in curtailing the risk connected with the Bayes estimator. This study aims to explore the utilization of two specific types of LFs, namely symmetric and asymmetric, to address the research objectives at hand.

### Squared error loss function

Gauss^30^ conducted a study on SELF as a symmetric LF, in which the study variable *X* and $\hat{\theta }$ were employed as estimators to estimate the unknown population parameter $\theta$. The expression for the SELF is provided below:

4 $$L\left( {\theta ,\hat{\theta }} \right) = \left( {\theta - \hat{\theta }} \right)^{2}$$

and the $\hat{\theta }_{{\left( {SELF} \right)}}$ is mathematized as

5 $$\hat{\theta }_{{\left( {SELF} \right)}} = E_{\theta /x} \left( \theta \right)$$

### Linex loss function (LLF)

The LLF is an asymmetric LF proposed by Varian^31^, which efficiently estimates the population parameter while mitigating the risks linked with the Bayes estimator. Mathematically, the LLF can be described as follows:

6 $$L\left( {\theta ,\hat{\theta }} \right) = \left( {e^{{c\left( {\theta - \hat{\theta }} \right)}} - c\left( {\theta - \hat{\theta }} \right) - 1} \right)$$

and $\hat{\theta }_{{\left( {LLF} \right)}}$ is defined as

7 $$\hat{\theta }_{{\left( {LLF} \right)}} = - \frac{1}{c}InE_{\theta /x} \left( {e^{ - c\theta } } \right)$$

## Ranked set sampling

Mclntyre^32^ initially introduced the concept of the RSS, which holds particular significance in cases where accurately measuring the study variable poses challenges. The estimator based on the RSS scheme is recognized for its superior efficiency when compared to simple random sampling (SRS). RSS combines the benefits of SRS using the additional sources of information, including auxiliary information, personal judgment, or expert knowledge. The comprehensive methodology for sample selection using the RSS scheme is elaborated upon within this context.

*Step 1* To implement the RSS scheme, the initial step involves identifying the $m^{2}$ units from the population under study. Subsequently, these units are randomly allocated into *m* sets of equal size, and all the units within the *m* sets are arranged in ascending order.

*Step 2* Once the units have been ranked, the selection process commences by choosing the first unit from the first set, the second unit from the second set, and so on, until the last unit is selected from the last set. This cycle of selecting units completes one iteration of the RSS with a size of *m*.

If required, the above steps can be repetitive *r* times to achieve the anticipated sample size of n = mr. This repetition ensures that the sample size reaches the intended value.

The mathematical description of the mean estimator based on the RSS scheme is as follows:

8 $$\overline{Z}_{{\left( {RSS} \right)}} = \frac{1}{m}\sum\limits_{i = 1}^{m} {Z_{i\left( i \right)} }$$

and variance

9 $${\text{var}} \left( {\overline{Z}_{{\left( {RSS} \right)}} } \right) = \frac{{\delta^{2} }}{m} - \frac{1}{{m^{2} }}\sum\limits_{i = 1}^{m} {\left( {\mu_{\left( i \right)} - \mu } \right)}$$

### Median ranked set sampling

Muttalk^33^ proposed the MRSS scheme as an altered rendition of RSS, with the objective of improving the estimation of the population mean. The succeeding two steps offers a comprehensive impression of the sample selection methodology utilized in MRSS:

*Step 1* Following a similar approach to RSS, the MRSS scheme involves identifying *m*^*2*^ units from the population under study. These units are subsequently allocated into *m* sets, each comprising m units of equal size. The units within each set are organized in ascending order.

*Step 2* After the ranking process is complete, if the sample size (represented as *m*) is an odd number, select the unit located at the $$\left\{ {{\raise0.7ex\hbox{${\left( {m + 1} \right)}$} \!\mathord{\left/ {\vphantom {{\left( {m + 1} \right)} 2}}\right.\kern-0pt} \!\lower0.7ex\hbox{$2$}}} \right\}$$ th position from each set. In this situation of an even sample size, choose the units ranked at the $$\left( {{\raise0.7ex\hbox{$m$} \!\mathord{\left/ {\vphantom {m 2}}\right.\kern-0pt} \!\lower0.7ex\hbox{$2$}}} \right)$$ th position from the first set and select the unit at the $$\left\{ {{\raise0.7ex\hbox{${\left( {m + 2} \right)}$} \!\mathord{\left/ {\vphantom {{\left( {m + 2} \right)} 2}}\right.\kern-0pt} \!\lower0.7ex\hbox{$2$}}} \right\}$$ th position from the last $$\left( {{\raise0.7ex\hbox{$m$} \!\mathord{\left/ {\vphantom {m 2}}\right.\kern-0pt} \!\lower0.7ex\hbox{$2$}}} \right)$$ th set. This series of steps constitutes a single cycle of the MRSS sample, with a size of *m*. To acquire an MRSS sample with a size of *n* = *mr,* the aforementioned steps can be repeated *r* times.

Applying MRSS, the mean estimator with single cycle, for an odd sample is expressed as follows:

10 $$\overline{Z}_{{\left( {MRSS} \right)O}} = \frac{1}{m}\left( {\sum\limits_{i = 1}^{m} {Z_{{i\left( {\frac{m + 1}{2}} \right)}} } } \right)$$

and variance

11 $${\text{var}} \left( {\overline{Z}_{{\left( {MRSS} \right)O}} } \right) = \frac{1}{m}\left( {\delta_{{\left( {\frac{m + 1}{2}} \right)}}^{2} } \right)$$

In the MRSS design, if the sample size is even, the population mean estimator for single cycle can be expressed as:

12 $$\overline{Z}_{{\left( {MRSS} \right)O}} = \frac{1}{m}\left( {\sum\limits_{i = 1}^{{{\raise0.7ex\hbox{$m$} \!\mathord{\left/ {\vphantom {m 2}}\right.\kern-0pt} \!\lower0.7ex\hbox{$2$}}}} {Z_{{i\left( \frac{m}{2} \right)}} } + \sum\limits_{i = 1}^{{{\raise0.7ex\hbox{$m$} \!\mathord{\left/ {\vphantom {m 2}}\right.\kern-0pt} \!\lower0.7ex\hbox{$2$}}}} {Z_{{\frac{m}{2} + i\left( {\frac{m + 1}{2}} \right)}} } } \right)$$

with variance

13 $${\text{var}} \left( {\overline{Z}_{{\left( {MRSS} \right)O}} } \right) = \frac{1}{m}\left( {\delta_{{\left( \frac{m}{2} \right)}}^{2} + \delta_{{\left( {\frac{m + 2}{2}} \right)}}^{2} } \right)$$

### Extreme ranked set sampling

A modified version of Ranked Set Sampling (RSS), known as the Extreme RSS (ERSS) design, was introduced by Samawi et al. ^34^. This modification is particularly beneficial when gathering a collection of units becomes more challenging than selecting extreme units alone. The authors provided a comprehensive explanation of the entire process involved in selecting an ERSS sample.

*Step 1* By randomly selecting *m*^*2*^ elements from the target population and distributing them into *m* sets, each of the same size, we ensure that the elements within each set are representative of the variable under consideration.

*Step 2* In the ERSS process, after ranking the units, the selection of extreme units depends on the sample size. If the sample size is even, the smallest unit from the first $$\left( {{\raise0.7ex\hbox{$m$} \!\mathord{\left/ {\vphantom {m 2}}\right.\kern-0pt} \!\lower0.7ex\hbox{$2$}}} \right)$$ th order set and the largest unit from the last $$\left( {{\raise0.7ex\hbox{$m$} \!\mathord{\left/ {\vphantom {m 2}}\right.\kern-0pt} \!\lower0.7ex\hbox{$2$}}} \right)$$ th order set are chosen.

However, when the sample size is odd, the ERSS scheme involves selecting the smallest unit from the first $$\left( {{\raise0.7ex\hbox{${m - 1}$} \!\mathord{\left/ {\vphantom {{m - 1} 2}}\right.\kern-0pt} \!\lower0.7ex\hbox{$2$}}} \right)$$ th order set, the largest unit from the last $$\left( {{\raise0.7ex\hbox{${m - 1}$} \!\mathord{\left/ {\vphantom {{m - 1} 2}}\right.\kern-0pt} \!\lower0.7ex\hbox{$2$}}} \right)$$ th order set, and the median unit from the last set. This completes one full cycle of the ERSS sampling method.

If deemed necessary, the above-mentioned two steps can be iterated r times to acquire an ERSS sample consisting of *n* = *mr* observations. When dealing with a uniform sample size in a single cycle, the mathematical representation for calculating the mean estimator of ERSS can be expressed as follows:

14 $$\overline{Z}_{{\left( {ERSS} \right)O}} = \frac{1}{m}\left( {\sum\limits_{i = 1}^{{\left( {\frac{m - 1}{2}} \right)}} {Z_{i\left( 1 \right)} } + \sum\limits_{i = 1}^{{\left( {\frac{m - 1}{2}} \right)}} {Z_{{\left( {\frac{m - 1}{2}} \right) + i\left( l \right)}} } + Z_{{m\left( {\frac{m + 1}{2}} \right)}} } \right)$$

and

15 $${\text{var}} \left( {\overline{Z}_{{\left( {ERSS} \right)O}} } \right) = \frac{1}{{2m^{2} }}\left( {\delta_{\left( 1 \right)}^{2} + \delta_{\left( m \right)}^{2} } \right) + \frac{1}{{l^{2} }}\left( {\delta_{{\left( {\frac{m + 1}{2}} \right)}}^{2} } \right)$$

For odd sample size, the estimator of ERSS in case of with one cycle is defined as

16 $$\overline{Z}_{{\left( {ERSS} \right)e}} = \frac{1}{m}\left( {\sum\limits_{i = 1}^{{\left( \frac{m}{2} \right)}} {Z_{i\left( 1 \right)} } + \sum\limits_{i = 1}^{{\left( \frac{m}{2} \right)}} {Z_{{\frac{m}{2} + i\left( l \right)}} } } \right)$$

with variance

17 $${\text{var}} \left( {\overline{Z}_{{\left( {ERSS} \right)e}} } \right) = \frac{1}{2m}\left( {\delta_{\left( 1 \right)}^{2} + \delta_{\left( m \right)}^{2} } \right)$$

## Measurement error

Measurement error refers to the variation between the observed value and the true value of a specific measurement. It is characterized by a constant magnitude that remains consistent across different observations. In this study, the covariate model has been employed to address ME. Additionally, to minimize the impact of ME, the multiple measurements technique has been utilized. This technique involves taking multiple measurements for each observation, allowing for a more precise estimation of the true underlying value.

### Using covariate model, EWMA CC with ME

The inspiration of ME on the Shewhart CC is examined by employing the model proposed by Bennett^35^. The model is defined as follows:

18 $$Y = X + \varepsilon$$

The covariate model assumes that the variable under study *X* follows a normal distribution with a mean of $\theta$ and a variance of $\delta^{2}$ for the in-control process. This model takes into account measurement inexactness by incorporating a random error term, $\varepsilon$. Linna and Woodall^10^ conducted further investigation on the covariate model, which is defined as follows:

19 $$Y = A + BX + \varepsilon$$

Assuming the known parameters involved in the model and the independence of X and $\varepsilon$, (i.e., $Cov\left( {X,\varepsilon } \right) = 0$), we consider the measured variable *Y*. *Y* is assumed to follow a normal distribution with a mean of *Aθ* + *B* and a variance of $B^{2} \delta^{2} + \delta_{m}^{2}$. *i.e.* $Y \sim N\left( {A + B\theta ,B^{2} \delta^{2} + \delta_{m}^{2} } \right)$. Based on these assumptions, the EWMA CC for the measured variable *Y* can be defined as follows:

20 $$F_{t} = \lambda \overline{y}_{t} + \left( {1 - \lambda } \right)F_{t - 1} ,\,and\,F_{0} = A + B\theta$$

Let $\overline{y}_{t}$ represent the sample mean for $t = 1,2,3,...$ , and smoothing constant $\lambda$. The control limits for EWMA CC based on covariate model are determined as follows:

21 $$UCL = \left( {A + B\theta } \right) + L\sqrt {\frac{{\lambda \left( {B^{2} \delta^{2} + \delta_{m}^{2} } \right)}}{{n\left( {2 - \lambda } \right)}}}$$

22 $$LCL = \left( {A + B\theta } \right) - L\sqrt {\frac{{\lambda \left( {B^{2} \delta^{2} + \delta_{m}^{2} } \right)}}{{n\left( {2 - \lambda } \right)}}}$$

### Under multiple measurements EWMA CC with ME

Linna and Woodall^10^ proposed a method, also adopted by Maravelakis et al.^12^ and Abbasi^36^ that is useful in minimaxing ME by taking multiple measurements instead of a single measurement per sampling unit. If the number of repeated measurements increases indefinitely, the variability of ME component tends to decrease towards zero. However, it should be noted that by increasing the number of measurements, the additional cost and time will be added at each additional measurement and these two factors cannot be ignored by the quality expert. Maravelakis et al.^12^ investigated how multiple measurements impact the effectiveness of the EWMA CC. They derived the plotting statistic specifically for the EWMA CC with multiple measurements as follows:

23 $$F_{t} = \lambda \overline{y} + \left( {1 - \lambda } \right)F_{t - 1} ,\,and\,F_{0} = A + B\theta$$

Assuming $\overline{y}$ represents the mean of multiple observations at time *t*, the control limits for the EWMA CC with multiple measurements can be described as follows:

24 $$UCL = \left( {A + B\theta } \right) + L\sqrt {\frac{\lambda }{{\left( {2 - \lambda } \right)}}\left( {\frac{{B^{2} \delta^{2} }}{n} + \frac{{\delta_{m}^{2} }}{nk}} \right)}$$

25 $$LCL = \left( {A + B\theta } \right) - L\sqrt {\frac{\lambda }{{\left( {2 - \lambda } \right)}}\left( {\frac{{B^{2} \delta^{2} }}{n} + \frac{{\delta_{m}^{2} }}{nk}} \right)}$$

where *k* is the number of measurements taken for the same sampling unit.

## Proposed Bayesian-EWMA CC based on with and without measurement error using various LFs under RSS schemes

In this section, we explore the utilization of various LFs within RSS schemes for the Bayesian-EWMA CC. The resulting P distribution, obtained through the implementation of a conjugate prior (normal prior), is presented as follows:

26 $$P\left( {\theta |y} \right) = \frac{1}{{\sqrt {2\pi } \sqrt {\frac{{\delta^{2} \delta_{0}^{2} }}{{\delta^{2} + n\delta_{0}^{2} }}} }}\exp \left[ { - \frac{1}{2}\left( {\frac{{\theta - \sum\limits_{i = 1}^{n} {\frac{{y_{i} \delta_{0}^{2} + \theta_{0} \delta_{0}^{2} }}{{\delta^{2} + n\delta_{0}^{2} }}} }}{{\sqrt {\frac{{\delta^{2} \delta_{0}^{2} }}{{\delta^{2} + n\delta_{0}^{2} }}} }}} \right)^{2} } \right]$$

the P distribution is normally distributed with mean $\theta_{n}$ and variance $\delta_{n}^{2}$ is given as $\theta /Y \sim N\left( {\theta_{n} ,\delta_{n}^{2} } \right)$, where $\theta_{n} = \frac{{n\overline{y} \delta_{0}^{2} + \delta^{2} \theta_{0} }}{{\delta^{2} + n\delta_{0}^{2} }}$ and $\delta_{n}^{2} = \frac{{\delta^{2} \delta_{0}^{2} }}{{\delta^{2} + n\delta_{0}^{2} }}$.

The suggested statistic for the EWMA CC based on Bayesian analysis under different RSS strategies is written as:

27 $$F_{t} = \lambda \hat{\theta }_{{ME\left( {RSS_{i} } \right)LF}} + \left( {1 - \lambda } \right)F_{t - 1}$$

where $$i = 1,2,3,$$ $$\begin{gathered} RSS_{1} = RSS \hfill \\ RSS_{2} = MRSS \hfill \\ RSS_{3} = ERSS \hfill \\ \end{gathered}$$, and $$F_{0} = 0$$.

### Using covariate model, proposed CC with ME using various RSS designs applying SELF for *P* distribution

The Bayes estimator utilizing Bayesian-EWMA CC, considering various RSS schemes under the SELF for P distribution, is as follows:

28 $$\hat{\theta }_{{psc\left( {SELF} \right)}} = \frac{{n\overline{y}_{{(RSS_{i} )}} \delta_{0}^{2} + \left( {B^{2} \delta^{2} + \delta_{m}^{2} } \right)\theta_{0} }}{{n\delta_{0}^{2} + B^{2} \delta^{2} + \delta_{m}^{2} }}$$

The asymptotic control limits for the EWMA CC, taking into account various RSS strategies under the assumption of SELF for the *P* distribution, are given by:

29 $$UCL = E\left( {\hat{\theta }_{{psc\left( {SELF} \right)}} } \right) + LS_{psc} \sqrt {\frac{\lambda }{2 - \lambda }}$$

30 $$LCL = E\left( {\hat{\theta }_{{psc\left( {SELF} \right)}} } \right) - LS_{psc} \sqrt {\frac{\lambda }{2 - \lambda }}$$

where $E\left( {\hat{\theta }_{{psc\left( {SELF} \right)}} } \right) = \frac{{n\left( {A + B\theta } \right)\delta_{0}^{2} + \left( {B^{2} \delta^{2} + \delta_{m}^{2} } \right)\theta_{0} }}{{n\delta_{0}^{2} + B^{2} \delta^{2} + \delta_{m}^{2} }}$ and $S_{psc} = \sqrt {\frac{{n\delta_{0}^{2} B^{2} \delta^{2} + \delta_{m}^{2} }}{{\left( {n\delta_{0}^{2} + B^{2} \delta^{2} + \delta_{m}^{2} } \right)^{2} }}}$ where $i = 1,2,3.$ $$\begin{gathered} RSS_{1} = RSS \hfill \\ RSS_{2} = MRSS \hfill \\ RSS_{3} = ERSS \hfill \\ \end{gathered}$$.

### Using multiple measurements method, Bayesian*-EWMA* CC with ME using various RSS strategies applying SELF for P distribution

The estimator for the EWMA CC using Bayesian methodology, considering distinct RSS strategies under the SELF for *P* distribution, is as follows:

31 $$\hat{\theta }_{{psmm\left( {SELF} \right)}} = \frac{{n\overline{y}_{{(RSS_{i} )}} \delta_{0}^{2} + \left( {\frac{{B^{2} \delta^{2} }}{n} + \frac{{\delta_{m}^{2} }}{nk}} \right)\theta_{0} }}{{n\delta_{0}^{2} + \left( {\frac{{B^{2} \delta^{2} }}{n} + \frac{{\delta_{m}^{2} }}{nk}} \right)}}$$

the asymptotic control limits for the recommended Bayesian CC, considering different RSS schemes using the SELF for *P* distribution with the multiple measurement’s method, are mathematized as follows:

32 $$UCL = E\left( {\hat{\theta }_{{psmm\left( {SELF} \right)}} } \right) + LS_{psmm} \sqrt {\frac{\lambda }{2 - \lambda }}$$

33 $$LCL = E\left( {\hat{\theta }_{{psmm\left( {SELF} \right)}} } \right) - LS_{psmm} \sqrt {\frac{\lambda }{2 - \lambda }}$$

where $E\left( {\hat{\theta }_{{psmm\left( {SELF} \right)}} } \right) = \frac{{n\left( {A + B\theta } \right)\delta_{0}^{2} + \left( {\frac{{B^{2} \delta_{{\left( {RSS} \right)_{i} }}^{2} }}{n} + \frac{{\delta_{m}^{2} }}{nk}} \right)\theta_{0} }}{{n\delta_{0}^{2} + \left( {\frac{{B^{2} \delta_{{\left( {RSS} \right)_{i} }}^{2} }}{n} + \frac{{\delta_{m}^{2} }}{nk}} \right)}}$ and $S_{psc} = \sqrt {\frac{{n\delta_{0}^{2} \left( {\frac{{B^{2} \delta_{{\left( {RSS} \right)_{i} }}^{2} }}{n} + \frac{{\delta_{m}^{2} }}{nk}} \right)}}{{\left( {n\delta_{0}^{2} + \left( {\frac{{B^{2} \delta^{2} }}{n} + \frac{{\delta_{m}^{2} }}{nk}} \right)} \right)^{2} }}}$, where $i = 1,2,3.$ $$\begin{gathered} RSS_{1} = RSS \hfill \\ RSS_{2} = MRSS \hfill \\ RSS_{3} = ERSS \hfill \\ \end{gathered}$$.

The Appendix A provides the remaining estimator, mean, standard deviation, and asymptotic control limits for the proposed Bayesian-EWMA CC based on ME. These estimates are based on different RSS designs under the assumption of LLF, while also incorporating an informative prior for both methods i.e., covariate model and multiple measurement of handling ME, and the complete r codes for evaluating the run length profile is included in Appendix B.

## Discussion on tables and main findings

Tables 1, 2 and 3 display the outcomes of the Bayesian EWMA CC with and without ME, considering three RSS schemes and two LFs for P and PP distribution using informative priors. Similarly, Tables 4, 5 and 6 follow the same pattern but incorporate multiple measurements of the same sampled values. In this section, the tables are examined, and the key findings of the offered EWMA CC applying Bayesian theory utilizing various RSS design are presented.

**Table 1:** The run length profiles for Bayesian-EWMA CC with ME using P and PP distribution under SELF for covariate model, for $\lambda$ = 0.25, *n* = 5.

Shift	Bayes-EWMA-RSS			Bayes-EWMA-MRSS			Bayes-EWMA-ERSS		
	ME = 0	ME = 0.5	ME = 1	ME = 0	ME = 0.5	ME = 1	ME = 0	ME = 0.5	ME = 1
0.0	371.34(367.87)	370.71(374.55)	369.87(372.12)	370.18(368.76)	369.78(375.34)	369.98(372.11)	371.01(368.98)	368.87(373.21)	371.01(373.78)
0.10	213.71(212.30)	263.30(256.76)	273.91(266.32)	194.28(192.31)	250.92(249.08)	261.04(254.44)	228.06(227.15)	267.87(256.05)	289.83(282.14)
0.20	86.62(84.06)	117.83(113.21)	144.56(137.47)	70.45(66.92)	106.45(101.82)	125.29(121.05)	98.59(96.41)	129.89(122.44)	166.69(157.85)
0.30	40.81(37.15)	61.34(57.62)	74.47(68.63)	31.19(27.48)	51.77(47.21)	64.80(59.91)	46.44(43.15)	71.88(67.38)	96.20(90.25)
0.40	21.77(18.64)	34.79(30.00)	44.98(39.90)	17.19(13.96)	28.48(24.28)	37.21(33.06)	26.17(22.86)	40.72(36.66)	55.84(51.99)
0.50	14.03(11.22)	21.90(17.62)	29.44(25.02)	10.85(8.22)	17.97(13.88)	23.02(18.86)	16.20(13.18)	25.89(21.66)	34.37(29.48)
0.60	9.42(6.87)	15.12(11.20)	20.00(15.72)	7.58(5.16)	12.53(8.78)	15.97(12.05)	11.07(8.24)	17.80(13.90)	23.81(19.87)
0.70	7.09(4.75)	11.08(7.50)	14.79(10.87)	5.76(3.59)	9.35(5.87)	11.89(8.29)	8.20(5.59)	13.14(9.47)	17.21(13.20)
0.80	5.53(3.47)	8.87(5.57)	11.40(7.76)	4.53(2.71)	7.33(4.24)	9.30(5.85)	6.31(4.08)	10.07(6.63)	13.25(9.49)
0.90	4.50(2.69)	7.22(4.16)	9.25(5.81)	3.69(2.09)	6.05(3.15)	7.50(4.37)	5.15(3.16)	8.20(4.89)	10.63(7.18)
1.0	3.78(2.17)	6.04(3.20)	7.68(4.47)	3.11(1.71)	5.10(2.44)	6.31(3.41)	4.26(2.49)	6.83(3.77)	8.76(5.40)
1.5	1.99(0.96)	3.37(1.27)	4.07(1.75)	1.71(0.77)	2.96(1.02)	3.50(1.35)	2.23(1.09)	3.72(1.46)	4.55(2.05)
2.0	1.37(0.56)	2.41(0.73)	2.83(0.95)	1.21(0.43)	2.16(0.60)	2.49(0.79)	1.49(0.63)	2.62(0.84)	3.10(1.13)
2.5	1.10(0.30)	1.94(0.53)	2.22(0.63)	1.04(0.19)	1.76(0.49)	1.99(0.53)	1.16(0.38)	2.08(0.57)	2.41(0.73)
3	1.01(0.12)	1.62(0.50)	1.87(0.50)	1(0)	1.44(0.50)	1.68(0.50)	1.04(0.20)	1.76(0.50)	2.00(0.54)

**Table 2:** Under covariate model, run length outcomes of the Bayesian-EWMA CC with ME using LLF for P distribution using $\lambda$ = 0.25, *n* = 5.

Shift	Bayes-EWMA-RSS			Bayes-EWMA-MRSS			Bayes-EWMA-ERSS		
	ME = 0	ME = 0.5	ME = 1	ME = 0	ME = 0.5	ME = 1	ME = 0	ME = 0.5	ME = 1
0.0	371.79(364.28)	370.21(365.43)	370.77(369.23)	370.77(368.76)	370.09(367.56)	369.89(366.55)	369.55(364.62)	369.76(364.21)	370.67(368.02)
0.10	214.20(207.72)	253.47(251.07)	274.99(269.89)	186.49(182.16)	228.03(227.51)	262.07(260.69)	220.53(217.98)	275.07(272.11)	290.82(288.66)
0.20	87.29(86.83)	117.20(115.43)	163.75(159.39)	66.78(64.33)	101.95(98.02)	127.96(126.72)	100.52(96.45)	141.96(138.87)	170.74(161.17)
0.30	39.62(36.14)	61.33(59.14)	80.79(75.58)	33.12(29.91)	48.38(45.50)	66.39(62.23)	37.49(36.71)	69.55(68.15)	94.53(92.64)
0.40	22.07(18.89)	34.75(31.19)	46.77(43.16)	17.53(14.40)	27.10(24.20)	37.13(34.16)	25.58(22.83)	39.25(36.23)	56.055(53.02)
0.50	13.91(11.12)	21.95(19.52)	28.95(25.91)	11.25(8.55)	16.56(13.60)	23.07(19.49)	15.71(12.69)	23.18(21.03)	33.83(30.90)
0.60	9.63(6.96)	14.48(11.54)	19.84(16.64)	7.78(5.44)	11.32(8.62)	15.75(12.78)	10.99(8.33)	16.91(13.79)	23.53(20.39)
0.70	7.13(4.76)	10.72(7.97)	14.31(11.60)	5.85(3.73)	8.41(5.96)	11.25(8.46)	8.08(5.63)	12.14(9.35)	16.76(13.72)
0.80	5.59(3.55)	8.08(5.66)	10.91(8.13)	4.59(2.74)	6.51(4.40)	8.78(6.09)	6.33(4.06)	9.34(6.73)	13.03(10.32)
0.90	4.49(2.66)	6.51(4.28)	8.53(5.96)	3.70(2.09)	5.26(3.24)	6.97(4.69)	5.04(3.10)	7.39(5.05)	10.17(7.68)
1.0	3.76(2.11)	5.34(3.34)	7.03(4.72)	3.12(1.68)	4.39(2.64)	5.67(3.58)	4.23(2.48)	6.08(3.94)	8.07(5.59)
1.5	2.00(0.96)	2.70(1.40)	3.42(1.85)	1.72(0.78)	2.29(1.14)	2.86(1.51)	2.23(1.09)	3.05(1.66)	3.88(2.18)
2.0	1.37(0.55)	1.77(0.81)	2.18(1.08)	1.22(0.43)	1.52(0.65)	1.84(0.86)	1.48(0.63)	1.97(0.94)	2.46(1.23)
2.5	1.10(0.31)	1.33(0.53)	1.61(0.71)	1.03(0.19)	1.19(0.41)	1.39(0.56)	1.16(0.38)	1.45(0.61)	1.77(0.81)
3	1.01(0.13)	1.12(0.33)	1.28(0.48)	1(0)	1.04(0.21)	1.14(0.36)	1.04(0.20)	1.19(0.41)	1.40(0.57)

**Table 3:** ARL and SDRL outcomes for Bayesian-EWMA CC under ME for PP distribution under LLF for covariate model, for $\lambda$ = 0.25, *n* = 5.

Shift	Bayes-EWMA-RSS			Bayes-EWMA-MRSS			Bayes-EWMA-ERSS		
	ME = 0	ME = 0.5	ME = 1	ME = 0	ME = 0.5	ME = 1	ME = 0	ME = 0.5	ME = 1
0.0	371.86(368.37)	371.08(368.55)	370.87(366.43)	370.89(368.30)	371.23(367.90)	370.09(368.67)	368.97(367.65)	370.89(368.36)	369.79(366.76)
0.10	202.83(198.65)	240.89(236.23)	246.28(238.10)	194.39(192.89)	235.50(233.58)	240.09(235.45)	234.57(236.80)	244.03(242.23)	265.90(261.54)
0.20	85.76(82.63)	120.36(118.31)	137.76(133.45)	71.03(64.97)	94.22(89.58)	122.84(118.13)	97.99(94.26)	140.46(136.55)	155.89(152.12)
0.30	38.60(34.53)	60.34(57.20)	69.74(66.68)	31.76(28.74)	48.57(45.53)	61.96(60.64)	48.21(44.69)	70.40(68.76)	112.78(110.12)
0.40	22.07(18.80)	33.91(30.72)	44.49(41.34)	17.17(14.01)	26.72(23.66)	36.14(32.87)	25.63(22.35)	40.37(36.98)	52.92(49.51)
0.50	13.76(10.87)	20.93(17.99)	27.89(25.09)	10.82(8.09)	16.51(13.27)	22.56(19.40)	16.45(13.24)	25.17(21.91)	33.55(31.30)
0.60	9.41(6.85)	14.26(11.40)	19.10(15.90)	7.60(5.24)	11.39(8.61)	15.20(12.36)	11.32(8.37)	16.95(13.80)	23.16(20.14)
0.70	7.11(4.79)	10.47(7.88)	14.025(11.10)	5.69(3.61)	8.43(6.02)	10.95(8.17)	8.22(5.66)	12.36(9.59)	16.72(13.51)
0.80	5.50(3.45)	8.15(5.68)	10.52(7.91)	4.46(2.67)	6.39(4.14)	8.49(5.97)	6.33(4.06)	9.40(6.92)	12.35(9.02)
0.90	4.50(2.71)	6.47(4.21)	7.86(5.16)	3.70(2.07)	5.26(3.27)	6.80(4.53)	5.10(3.14)	7.31(4.95)	9.31(6.38)
1.0	3.75(2.14)	5.34(3.29)	6.34(3.89)	3.10(1.66)	4.34(2.55)	5.57(3.46)	4.26(2.54)	6.08(3.93)	7.55(4.96)
1.5	1.99(0.95)	2.71(1.42)	3.10(1.59)	1.70(0.78)	2.26(1.13)	2.80(1.48)	2.21(1.09)	3.05(1.66)	3.56(1.87)
2.0	1.36(0.55)	1.77(0.81)	1.97(0.90)	1.20(0.42)	1.51(0.65)	1.83(0.84)	1.50(0.64)	1.97(0.93)	2.23(1.05)
2.5	1.09(0.30)	1.34(0.53)	1.45(0.60)	1.03(0.19)	1.18(0.41)	1.38(0.56)	1.16(0.38)	1.47(0.61)	1.62(0.69)
3	1.01(0.12)	1.12(0.33)	1.17(0.39)	1(0)	1.04(0.21)	1.14(0.36)	1.04(0.20)	1.20(0.41)	1.28(0.48)

**Table 4:** Utilizing SELF, the run length profile values of the Bayesian-EWMA CC in presence ME for P and PP distribution for multiple measurements, for $\lambda$ = 0.25, *n* = 5.

Shift	Bayes-EWMA-RSS			Bayes-EWMA-MRSS			Bayes-EWMA-ERSS		
	ME = 0	ME = 0.5	ME = 1	ME = 0	ME = 0.5	ME = 1	ME = 0	ME = 0.5	ME = 1
0.0	371.34(367.87)	370.11(373.10)	370.95(368.53)	370.18(368.76)	369.23(366.22)	371.03(369.06)	371.01(368.98)	370.02(366.76)	369.88(366.53)
0.10	213.71(212.30)	223.04(226.47)	229.05(222.84)	194.28(192.31)	202.76(197.20)	219.12(213.44)	228.06(227.15)	236.84(234.76)	240.56(236.65)
0.20	86.62(84.06)	96.54(92.55)	100.27(97.50)	70.45(66.92)	77.64(70.47)	84.81(82.11)	98.59(96.41)	108.15(105.58)	117.78(113.18)
0.30	40.81(37.15)	46.31(42.22)	47.88(43.27)	31.19(27.48)	35.86(31.40)	39.81(35.34)	46.44(43.15)	51.75(47.11)	58.03(53.72)
0.40	21.77(18.64)	25.48(21.08)	26.97(22.88)	17.19(13.96)	20.85(16.83)	22.46(18.41)	26.17(22.86)	30.66(25.33)	33.18(27.94)
0.50	14.03(11.22)	16.17(12.47)	17.20(13.17)	10.85(8.22)	12.76(9.12)	14.06(10.16)	16.20(13.18)	19.64(15.03)	21.44(16.72)
0.60	9.42(6.87)	11.29(7.64)	12.16(8.56)	7.58(5.16)	9.12(5.75)	10.06(6.41)	11.07(8.24)	13.09(9.29)	15.54(12.27)
0.70	7.09(4.75)	8.50(5.29)	8.92(5.47)	5.76(3.59)	6.91(3.84)	7.54(4.45)	8.20(5.59)	9.72(6.23)	9.35(6.04)
0.80	5.53(3.47)	6.76(3.80)	7.16(4.13)	4.53(2.71)	5.51(2.81)	6.08(3.21)	`6.31(4.08)	7.62(4.44)	8.29(5.00)
0.90	4.50(2.69)	5.55(2.76)	5.90(3.10)	3.69(2.09)	4.73(2.17)	5.03(2.39)	5.15(3.16)	6.31(3.39)	6.69(3.66)
1.0	3.78(2.17)	4.76(2.20)	5.04(2.38)	3.11(1.71)	4.06(1.72)	4.32(1.88)	4.26(2.49)	5.35(2.59)	5.69(2.90)
1.5	1.99(0.96)	2.79(0.93)	2.93(1.03)	1.71(0.77)	2.46(0.76)	2.59(0.83)	2.23(1.09)	3.07(1.08)	3.23(1.17)
2.0	1.37(0.56)	2.07(0.57)	2.13(0.60)	1.21(0.43)	1.86(0.50)	1.95(0.52)	1.49(0.63)	2.21(0.62)	2.32(0.68)
2.5	1.10(0.30)	1.68(0.50)	1.75(0.50)	1.04(0.19)	1.48(0.40)	1.55(0.48)	1.16(0.38)	1.81(0.50)	1.88(0.50)
3	1.01(0.12)	1.35(0.47)	1.41(0.49)	1(0)	1.17(0.27)	1.23(0.38)	1.04(0.20)	1.49(0.45)	1.57(0.51)

**Table 5:** The run length profile results for Bayesian-EWMA CC with ME for P distribution under LLF for multiple measurements method, for $\lambda$ = 0.25, *n* = 5.

Shift	Bayes-EWMA-RSS			Bayes-EWMA-MRSS			Bayes-EWMA-ERSS		
	ME = 0	ME = 0.5	ME = 1	ME = 0	ME = 0.5	ME = 1	ME = 0	ME = 0.5	ME = 1
0.0	371.79(364.28)	370.09(367.77)	370.56(367.78)	370.77(368.76)	370.51(369.88)	370.01(368.67)	369.55(364.62)	369.87(365.43)	370.01(367.87)
0.10	214.20(207.72)	233.78(231.11)	241.11(244.84)	186.49(182.16)	220.67(218.43)	238.95(236.87)	220.53(217.98)	233.88(230.17)	258.13(253.41)
0.20	87.29(86.83)	98.57(95.24)	102.23(99.09)	66.78(64.33)	85.81(82.54)	93.38(86.80)	100.52(96.45)	110.60(109.95)	134.17(132.77)
0.30	39.62(36.14)	45.62(43.16)	49.45(47.01)	33.12(29.91)	35.05(32.46)	39.01(36.44)	37.49(36.71)	52.27(48.39)	67.29(63.85)
0.40	22.07(18.89)	25.14(22.21)	27.49(24.23)	17.53(14.40)	19.02(15.76)	21.49(18.50)	25.58(22.83)	33.87(29.91)	37.03(33.48)
0.50	13.91(11.12)	15.73(12.77)	16.96(14.15)	11.25(8.55)	12.10(9.37)	13.28(10.13)	15.71(12.69)	20.19(17.19)	23.95(18.57)
0.60	9.63(6.96)	10.57(7.73)	11.74(8.90)	7.78(5.44)	8.35(5.79)	9.30(6.74)	10.99(8.33)	13.49(10.44)	15.46(12.04)
0.70	7.13(4.76)	7.85(5.34)	8.51(6.02)	5.85(3.73)	6.26(4.14)	6.81(4.53)	8.08(5.63)	8.97(6.45)	11.01(7.95)
0.80	5.59(3.55)	6.12(3.91)	6.58(4.28)	4.59(2.74)	4.92(3.04)	5.33(3.36)	6.33(4.06)	7.58(5.15)	8.27(5.63)
0.90	4.49(2.66)	4.92(3.06)	5.30(3.27)	3.70(2.09)	4.03(2.34)	4.31(2.54)	5.04(3.10)	5.97(3.73)	6.48(4.17)
1.0	3.76(2.11)	4.14(2.44)	4.46(2.64)	3.12(1.68)	3.35(1.85)	3.59(2.01)	4.23(2.48)	4.92(2.99)	5.29(3.20)
1.5	2.00(0.96)	2.16(1.05)	2.31(1.14)	1.72(0.78)	1.82(0.84)	1.94(0.91)	2.23(1.09)	2.51(1.24)	2.66(1.35)
2.0	1.37(0.55)	1.45(0.61)	1.54(0.67)	1.22(0.43)	1.26(0.48)	1.33(0.53)	1.48(0.63)	1.65(0.72)	1.75(0.78)
2.5	1.10(0.31)	1.15(0.36)	1.19(0.41)	1.03(0.19)	1.05(0.23)	1.09(0.28)	1.16(0.38)	1.26(0.47)	1.31(0.50)
3	1.01(0.13)	1.03(0.17)	1.05(0.22)	1(0)	1(0)	1(0)	1.04(0.20)	1.07(0.26)	1.10(0.31)

**Table 6:** Under LLF, ARL and SDRL results for Bayesian-EWMA CC in presence of ME for PP distribution for multiple measurements method, for $\lambda$ = 0.25, *n* = 5.

Shift	Bayes-EWMA-RSS			Bayes-EWMA-MRSS			Bayes-EWMA-ERSS		
	ME = 0	ME = 0.5	ME = 1	ME = 0	ME = 0.5	ME = 1	ME = 0	ME = 0.5	ME = 1
0.0	371.86(368.37)	369.67(367.21)	370.45(365.34)	370.89(368.30)	369.11(367.87)	369.89(367.21)	368.97(367.65)	371.65(368.22)	370.67(368.43)
0.10	202.83(198.65)	220.17(215.18)	240.93(238.69)	194.39(192.89)	191.98(188.68)	199.12(197.34)	234.57(236.80)	249.02(248.78)	258.07(253.48)
0.20	85.76(82.63)	92.79(88.98)	102.75(101.01)	71.03(64.97)	79.11(76.94)	108.51(105.21)	97.99(94.26)	105.88(102.92)	115.05(112.99)
0.30	38.60(34.53)	44.13(40.74)	49.13(46.80)	31.76(28.74)	35.69(33.01)	45.34(42.13)	48.21(44.69)	52.81(49.82)	60.34(56.89)
0.40	22.07(18.80)	24.79(21.72)	27.06(24.01)	17.17(14.01)	19.08(15.90)	31.23(30.21)	25.63(22.35)	29.71(26.52)	33.74(30.13)
0.50	13.76(10.87)	15.53(12.53)	16.82(13.85)	10.82(8.09)	12.00(9.16)	19.77(16.19)	16.45(13.24)	18.19(15.49)	20.13(17.08)
0.60	9.41(6.85)	10.45(7.79)	11.75(9.22)	7.60(5.24)	8.36(5.91)	12.62(9.20)	11.32(8.37)	12.55(9.81)	13.61(10.69)
0.70	7.11(4.79)	7.86(5.34)	8.39(5.88)	5.69(3.61)	6.35(4.13)	9.09(6.10)	8.22(5.66)	9.01(6.41)	9.99(7.19)
0.80	5.50(3.45)	6.05(3.91)	6.53(4.32)	4.46(2.67)	4.90(2.99)	6.81(4.19)	6.33(4.06)	7.90(5.46)	7.67(6.27)
0.90	4.50(2.71)	4.89(3.01)	5.31(3.34)	3.70(2.07)	3.97(2.28)	5.41(3.15)	5.10(3.14)	6.91(4.60)	6.09(5.34)
1.0	3.75(2.14)	4.05(2.36)	4.39(2.58)	3.10(1.66)	3.36(1.85)	4.44(2.44)	4.26(2.54)	4.69(2.81)	5.02(3.92)
1.5	1.99(0.95)	2.14(1.04)	2.30(1.14)	1.70(0.78)	1.81(0.83)	2.27(1.05)	2.21(1.09)	2.40(1.21)	2.57(3.06)
2.0	1.36(0.55)	1.45(0.61)	1.53(0.66)	1.20(0.42)	1.27(0.48)	1.50(0.62)	1.50(0.64)	1.59(0.70)	1.68(1.34)
2.5	1.09(0.30)	1.15(0.36)	1.20(0.41)	1.03(0.19)	1.06(0.24)	1.15(0.36)	1.16(0.38)	1.22(0.44)	1.28(0.76)
3	1.01(0.12)	1.03(0.17)	1.05(0.22)	1(0)	1(0)	1.02(0.17)	1.04(0.20)	1.06(0.25)	1.09(0.48)

Tables 1, 2, 3, 4, 5 and 6 reveal that ARL and SDRL values are decreased as the shift is increased from 0.10 to 0.20 and so on up till 4. Every minor to moderate shift in the process parameter detects earlier as the ARL for each shift is decreased as compared with the earlier ARL value which approaches unit value at shift 4. These phenomena can be observed for no error, error of 0.5, or 1 under all the three Bayesian RSS, MRSS, and ERSS techniques in all the six tables which is proved as the basic quality of EWMA CCs. Upon examining the tables concerning the influence of measurement error on CC efficiency, we observe a consistent pattern across all tables. As the error magnitude increases from zero to 0.5 and subsequently to a unit value, the ARLs also increase correspondingly. This leads to a delay in detecting process shifts for all types of RSS. This trend leads us to highlight that the ME has negative effect on the efficiency of EWMA CCs for identifying of moderate to minor process shifts in the industrial production. If we examine Table 1, we can observe the results for the run length profile of the proposed Bayesian-EWMA CC. The table displays these outcomes for distinct RSS designs applying SELF for the covariate model, taking into account *A* = *0* and *B* = *1*. Additionally, the table presents results for different values of $\frac{{\delta_{m}^{2} }}{{\delta^{2} }}$, representing no error, 0.5, and 1. The run length values increased with an increase in the value of $\frac{{\delta_{m}^{2} }}{{\delta^{2} }}$. For example, at $\frac{{\delta_{m}^{2} }}{{\delta^{2} }}$ = 0.0, 0.5 and 1 with $\lambda = 0.25$ and shift $\sigma = 0.40$, the ARL values are the 21.77, 34.79 and 44.98 for RSS, 17.19, 28.48 and 37.21 for MRSS while for ERSS are 26.17, 40.72 and 55.84. The same can be seen from Table 6 at $\frac{{\delta_{m}^{2} }}{{\delta^{2} }}$ = 0.0, 0.5 and 1 with $\lambda = 0.25$ and $\sigma = 0.40$, the ARL values are 22.07, 24.79 and 27.06 for RSS, while 17.17, 19.08 and 31.23 for MRSS and for ERSS are 25.63, 29.71 and 33.74.

Another aspect of these tables can also be elaborated that ARL values of the Bayesian MRSS technique are smaller than the RSS and ERSS techniques for all the tables for any shift which indicates that the MRSS technique is efficient as compared with other two techniques of ranked set sampling and it performs better under the ME problem. It is also clearly seen from above mentioned values of Table 1. We can also see the same trend from Table 3. Table 3 displays the effectiveness evaluation of the suggested CC applying ME utilizing LLF. The table examines distinct RSS designs for PP distribution while considering changes in the values of $\frac{{\delta_{m}^{2} }}{{\delta^{2} }}$ at *λ* = 0.25. For example, at $\frac{{\delta_{m}^{2} }}{{\delta^{2} }}$ = 0.0, 0.5 and 1 with $\sigma = 0.30$, the ARL outcomes are 38.60, 60.34 and 69.74 for RSS, 31.76, 48.57 and 61.96 for MRSS. The ARL values for ERSS are 48.21, 70.40 and 112.78.

When we compare the Tables 1, 2 and 3 with the Tables 4, 5 and 6 respectively, it can be observed that corresponding tables are the same except later ones are constructed with multiple measurements having all other features same as those of first three tables except “no error” columns of Tables 4, 5 and 6 are also same as were in Tables 1, 2 and 3. Tables 4, 5 and 6 reveal that the multiple measurements of the same sample play an important role to reduce the ME effect. The ARL values of Tables 4, 5 and 6 are comparatively smaller than the respective values under the Tables 1, 2 and 3 to show that multiple measurements reduce the effect of ME and increase the chart efficiency to detect the process shift earlier. For example, Table 4 shows that at $\frac{{\delta_{m}^{2} }}{{\delta^{2} }}$ = 0.5 and 1 with $\sigma = 0.40$, the ARL values are 25.48 and 26.97 for RSS, 20.85 and 22.46 applying MRSS and ARL outcomes for ERSS are 30.66 and 33.18 which are far less than the corresponding values of Table 1 explained in earlier discussion.

On the basis of above discussion, we can devise main findings here.

- The EWMA CCs are efficient in detection of moderate to minor process shifts as shown from ARL and SDRL values in all the six tables for proposed charts.
- That ME has negative effect on the efficiency of the recommended CCs which is also discussed above.
- The MRSS performs better than other two i.e., RSS and ERSS even during the problem of ME for detection of process shifts earlier. The same is clear from all the ARL values shown in the tables constructed for proposed CCs.
- The multiple measurements reduce the error effect as is clear from ARL values of Tables 4, 5 and 6 and discussion on tables earlier. It is proved that multiple measures reduce the error effect for our proposed charts.
- Based on the analysis of P and PP distributions in the Bayesian framework, it can be observed that the offered Bayesian EWMA CC in the existence ME, implemented under the MRSS scheme, demonstrates reduced sensitivity to ME compared to other RSS designs. These observations are derived from the utilization of informative priors and the consideration of both LFs.

## Real life data applications

In this article, we showcase the application of the proposed Bayesian-EWMA CC with ME using data obtained from Montgomery ^37^ in the context of the hard-bake process in semiconductor production. The dataset consists of 45 samples, where each sample consists of 5 wafers, resulting in a total of 225 data points. The measurements of the flow width are recorded in microns, with a fixed time interval of one hour between each sample. The first 30 samples, comprising 150 observations, are classified as the under-control process (referred to as the phase-I dataset). Conversely, the remaining 15 samples, totaling 75 observations, are considered the out-of-control process (referred to as the phase-II dataset).

**Figure 1:**
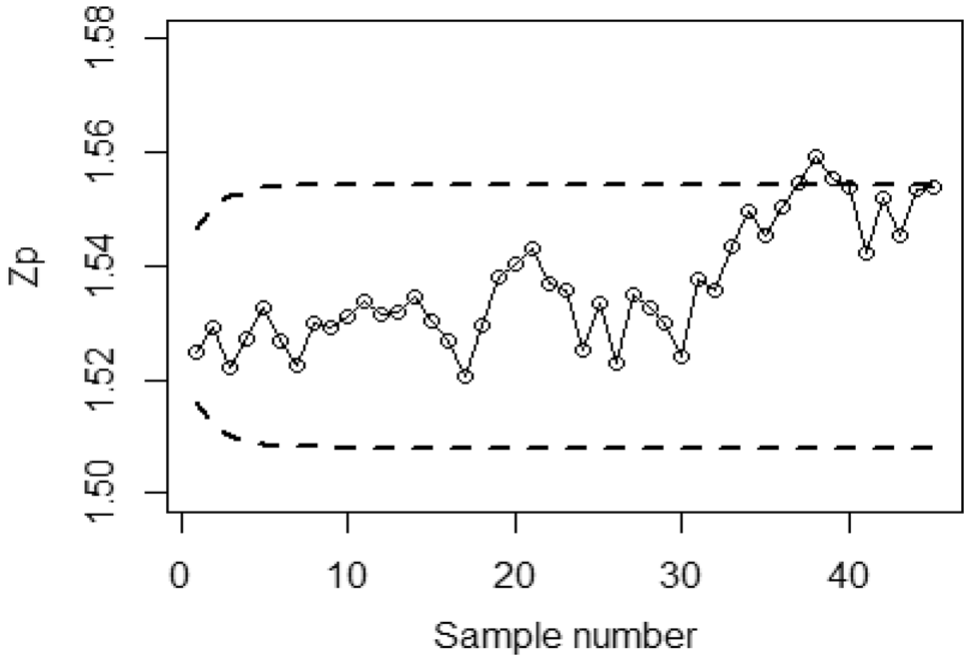
Bayesian-EWMA CC with RSS by utilizing SELF for covariate model at $\frac{{\delta_{m}^{2} }}{{\delta^{2} }} = 0$.

**Figure 2:**
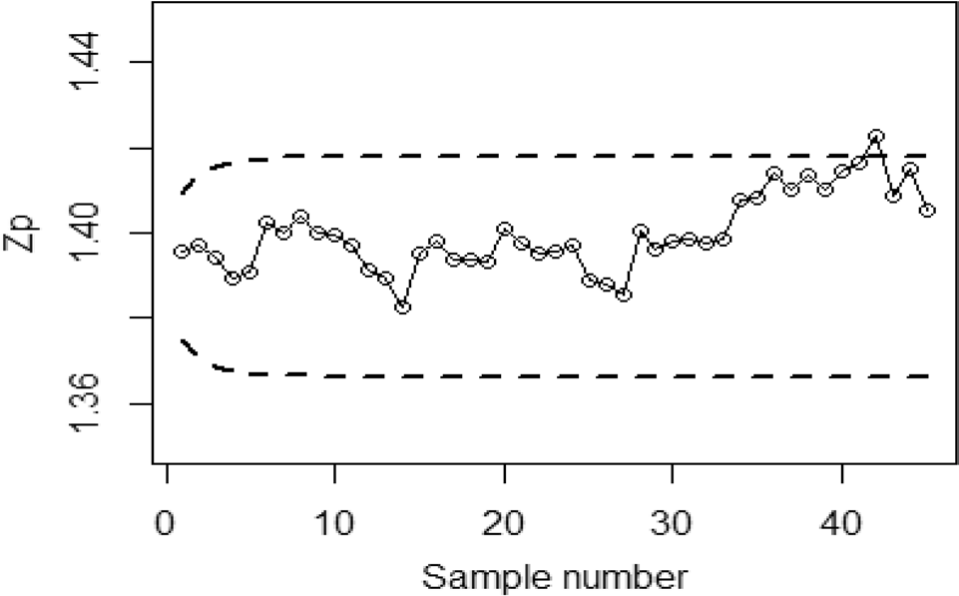
Utilizing SELF, Bayesian CC by utilizing RSS for covariate model at $\frac{{\delta_{m}^{2} }}{{\delta^{2} }} = 0.5.$

**Figure 3:**
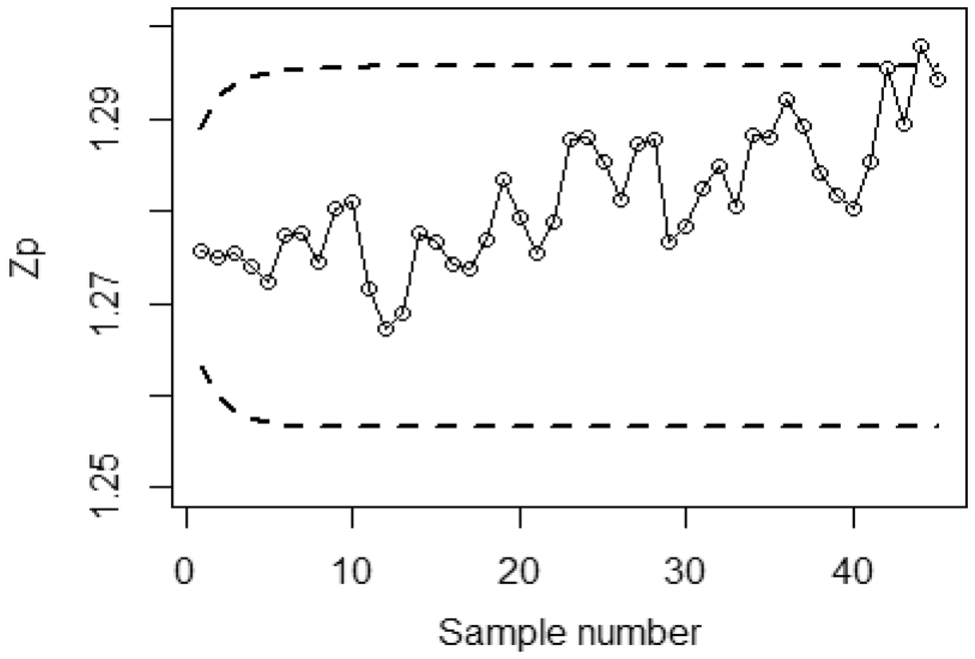
*Bayesian-EWMA* CC for covariate model using RSS design with SELF at $\frac{{\delta_{m}^{2} }}{{\delta^{2} }} = 1.$

To implement the proposed Bayesian-EWMA CC under covariate model utilizing RSS strategies with SELF, we consider various values of the error ratio $\frac{{\delta_{m}^{2} }}{{\delta^{2} }}$, specifically 0.0, 0.5, and 1 Figs. 1, 2, and 3 present the outcomes of the offered CC for the covariate model under SELF applying RSS. The values of $\frac{{\delta_{m}^{2} }}{{\delta^{2} }}$ considered are 0.0, 0.5, and 1, respectively. Based on the analysis, it is observed that the process deviates from control in the 36th, 41st, and 43rd samples.

**Figure 4:**
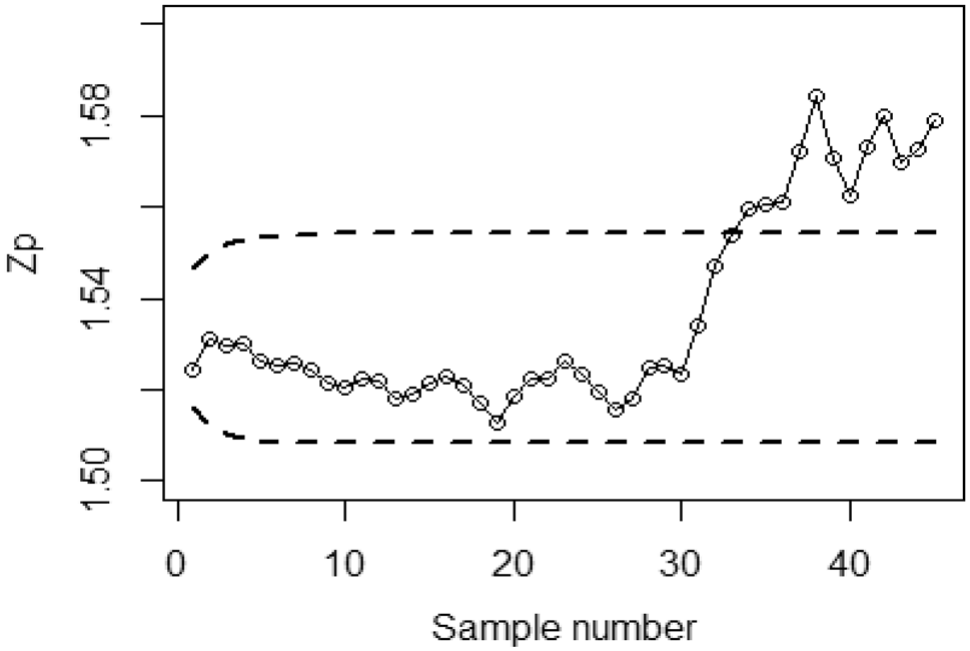
Utilizing SELF, *Bayesian-EWMA* CC for covariate model with MRSS for $\frac{{\delta_{m}^{2} }}{{\delta^{2} }} = 0$.

**Figure 5:**
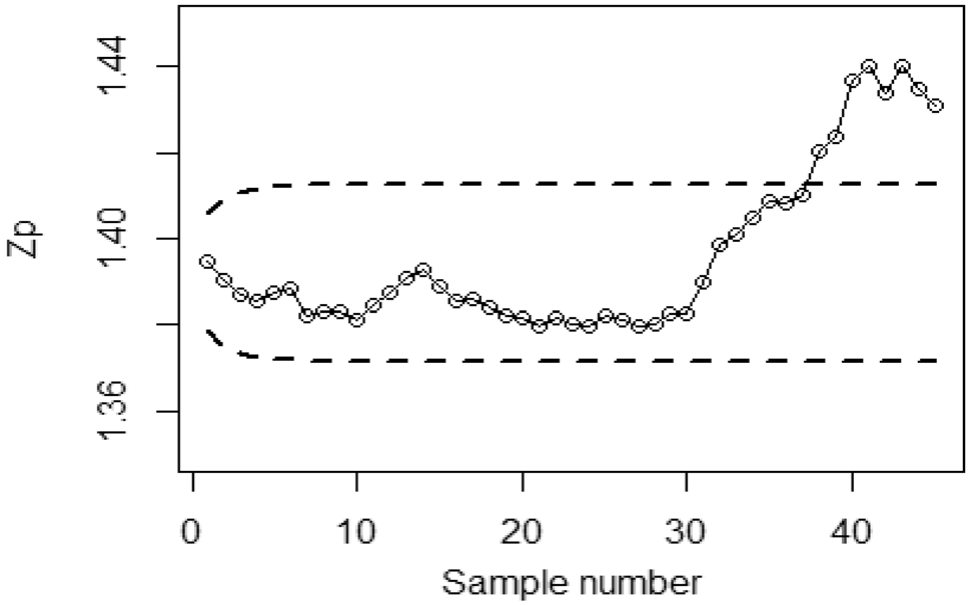
*Bayesian-EWMA* CC with covariate model under MRSS utilizing SELF for $\frac{{\delta_{m}^{2} }}{{\delta^{2} }} = 0.5.$

**Figure 6:**
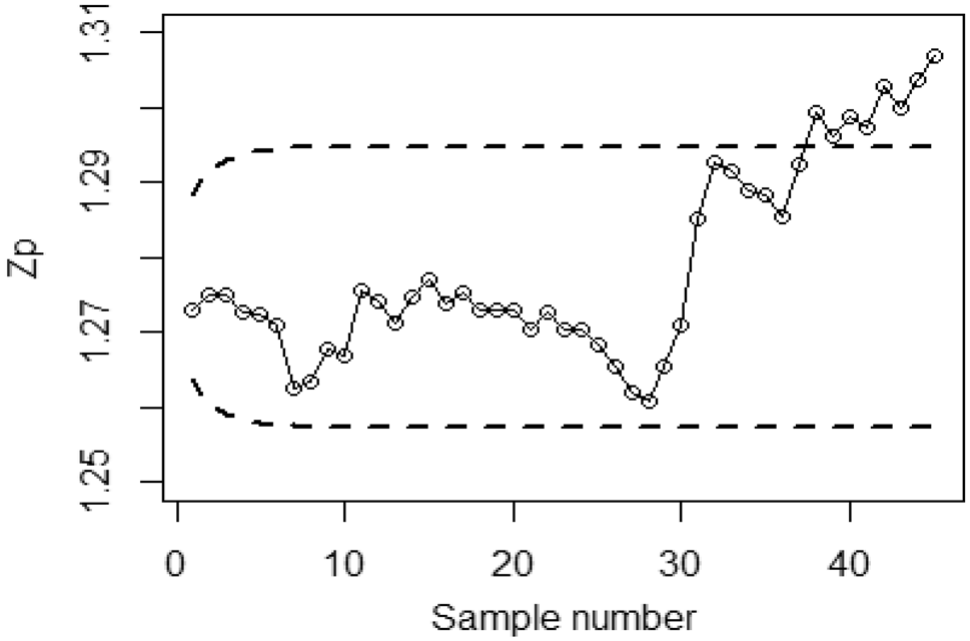
*Bayesian-EWMA* CC for covariate model with MRSS by using SELF for $\frac{{\delta_{m}^{2} }}{{\delta^{2} }} = 1.$

**Figure 7:**
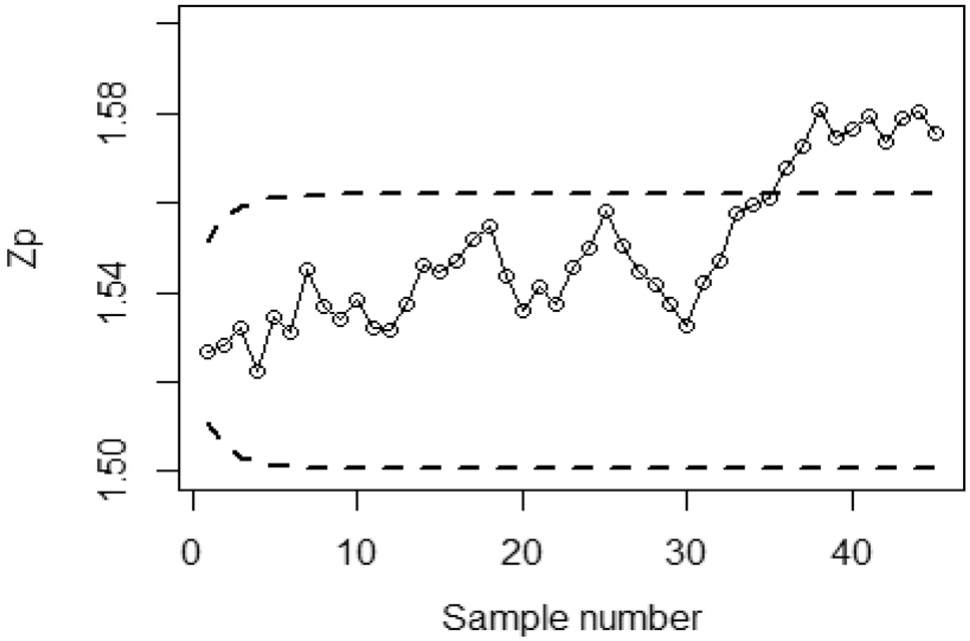
*Bayesian-EWMA* CC using ERSS for covariate model by using SELF for $\frac{{\delta_{m}^{2} }}{{\delta^{2} }} = 0$.

**Figure 8:**
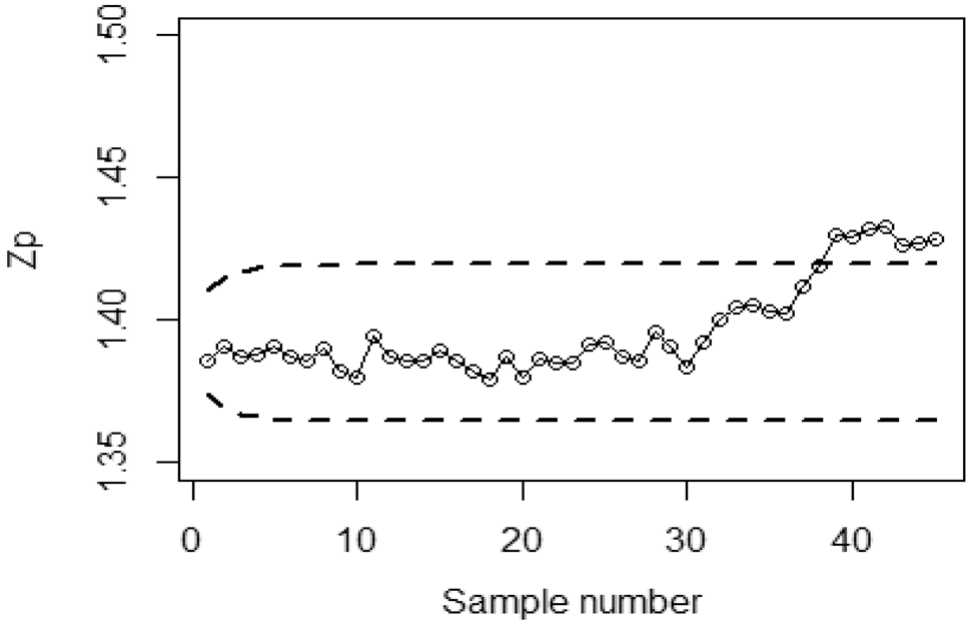
Bayesian EWMA CC under ERSS for covariate model by using SELF for $\frac{{\delta_{m}^{2} }}{{\delta^{2} }} = 0.5.$

**Figure 9:**
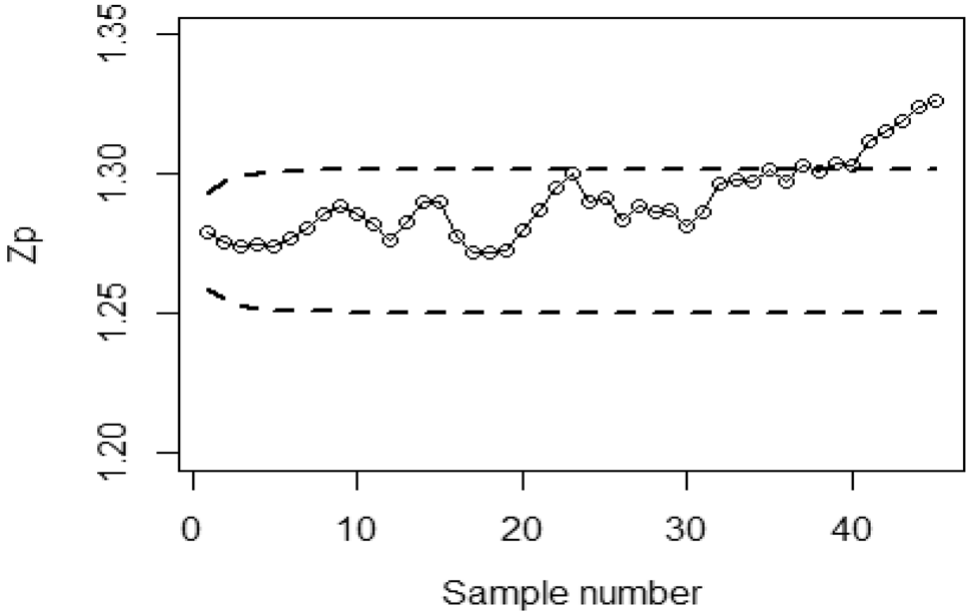
Utilizing covariate model, Bayesian CC under ERSS using SELF for $\frac{{\delta_{m}^{2} }}{{\delta^{2} }} = 1.$

Figures 4, 5, and 6 demonstrate the implementation of the proposed CC using the MRSS designs, employing the SELF with a covariate model. The chart considers outcomes of and error ratios $\frac{{\delta_{m}^{2} }}{{\delta^{2} }}$ equal to 0.0, 0.5, and 1. Based on these figures, it is evident that the process shows out-of-control signals in the 32nd, 36th, and 38th samples. Similarly, Figs. 7, 8, and 9 illustrate the performance of the proposed CC under the ERSS design, indicating out-of-control signals in the 35th, 38th, and 40th samples within the same scenario. This highlights that the MRRS scheme is most suitable as compared with other two schemes regarding efficiency of control charts for process shift detection during industrial manufacturing.

## Conclusion

This article examines the impact of ME on the EWMA CC utilizing Bayesian methodologies when utilizing distinct RSS designs applying LFs, specifically SELF and LLF. The effectiveness of the suggested CC with ME is assessed through the evaluation of the ARL and SDRL. The ARL values provide insights into the simulation results of Bayesian-EWMA CCs using RSS schemes for both the covariate method and multiple measurements. Our findings indicate that the proposed Bayesian-EWMA CC, implemented with the MRSS scheme, demonstrates improved efficiency compared to other RSS schemes in the presence of ME. Based on these results, we recommend employing the Bayesian-EWMA-MRSS CC for effective monitoring of process mean shifts in the presence of ME.

## Limitations of the study

When faced with a large sample size, computing the Bayesian EWMA CC with ME under RSS strategies can be complex. The Bayesian updating process entails calculating the distribution for both the process mean and variance at each sample point, which can be time-consuming and demanding in terms of resources. Additionally, the Bayesian approach necessitates the careful specification of prior distributions for the process mean and variance. If these priors are not chosen with care, they can negatively impact the performance of the CC. Furthermore, selecting appropriate prior distributions often involves subjectivity and relies on expert knowledge, introducing the potential for bias in the analysis.

## Future recommendation

The Bayesian EWMA CC with ME and RSS designs has the potential for broader application beyond its original context. It can serve as a foundation for developing other CCs that incorporate memory. Additionally, this approach shows promise in accommodating distributions beyond the normal distribution. For instance, it can be adapted to handle data that follows a Binomial or Poisson distribution. In such cases, adjustments to the likelihood function used in Bayesian updating may be necessary. Expanding the proposed approach to encompass a variety of CCs and non-normal distributions presents an opportunity to enhance the efficiency and effectiveness of quality control processes in various industries, including finance, healthcare, and production.

### Supplementary Information

Supplementary Information.

## Data Availability

The corresponding author can provide the datasets utilized and/or examined during the present study upon a reasonable request.
