# Supplementary material for: Performance of Bayesian EWMA control chart with measurement error under ranked set sampling schemes with application in industrial engineering
Source: Sci Rep. 2023 Aug 28;13:14042. doi: 10.1038/s41598-023-40656-x (PMC10462758; doi:10.1038/s41598-023-40656-x)
Supplement: Supplementary file 1 — Supplementary Information. [file 41598_2023_40656_MOESM1_ESM.docx]

**Appendix A**

Bayesian EWMA CC with ME applying distinct LFs using RSS strategies.

| Bayesian EWMA CC utilizing various LFs using RSS designs | Estimator of $\theta$ for the Bayesian CC | Upper control limits of Bayesian EWMA CC | Lower control limits of Bayesian EWMA CC |
| --- | --- | --- | --- |
| Bayesian EWMA CC for covariate model in the existence of ME applying LLF utilizing RSS strategies for P distribution |  | , where    and $S_{plc}=\sqrt{\frac{n\delta_{0}^{2}B^{2}\delta^{2}+\delta_{m}^{2}}{\left(n\delta_{0}^{2}+B^{2}\delta^{2}+\delta_{m}^{2}\right)^{2}}}$  |  |
| Bayesian EWMA CC for covariate model with ME utilizing LLF applying RSS designs for PP distribution |  | , where    and  $S_{pplc}=\sqrt{\frac{n\delta_{0}^{2}B^{2}\delta^{2}+\delta_{m}^{2}}{\left(n\delta_{0}^{2}+B^{2}\delta^{2}+\delta_{m}^{2}\right)^{2}}}$. |  |
| Bayesian EWMA CC for multiple measurements method with ME under LLF applying RSS designs for P distribution |  | , where    and  . |  |
| Bayesian EWMA CC for multiple measurements method based on ME utilizing LLF applying RSS strategies for PP distribution |  | , where   |  |

**Appendix B**

#ARL using posterior distribution under SELF

#For RSS

rm(list=ls(all=TRUE))

library(MASS)

n=m=5;

est_rss=c();rssx=matrix(,m,m);

Mx=c(); Zp=c(); ucl=c(); lcl=c(); rl=c();

mu=0; sig=1;

rssx=matrix(,m,m);

sigM = 0.2724601

ld=0.25; L=2.9142; A=0;

B=1;sigyy=1; delta = 0.0; sigm = 0.30 ;muy=0;

# Baysian Part

m_pr=0;sd_pr=1

m_po=A+B*muy

vr=(B^2*sig^2+sigm)

# Under Self

NU=(n*m_po*(sd_pr^2)+(vr)*m_pr)

DE=vr+n*sd_pr^2

muM=NU/DE

sig=1

sigma=(n^2*sigM^2*(sd_pr)^4)/DE^2

shift=A+B*(muy+delta*sqrt(1/n))

for(j in 1:10000)

{

for(i in 1:100000)

{

for(k in 1:m)

{

x=rnorm(m,shift,sqrt(vr))

dat=data.frame(x)

xs=dat[order(x),]

rssx[k,]=xs

}

mrssx=c(rssx[1,3],rssx[2,3],rssx[3,3],rssx[4,3],rssx[5,3])

y=mean(mrssx)

NUs=(n*mean(y)*(sd_pr)+(vr)*m_pr)

DEs=vr+n*sd_pr^2

Mx[i]=NUs/DEs

if(i==1)

{Zp[i]=ld*Mx[i]+ (1-ld)*muM;}

else{Zp[i]=ld*Mx[i]+(1-ld)*Zp[i-1];}

ucl[i]=muM+L*sqrt(sigma)*sqrt((ld/(2-ld))*(1-(1-ld)^(2*i)))

lcl[i]=muM-L*sqrt(sigma)*sqrt((ld/(2-ld))*(1-(1-ld)^(2*i)))

if(Zp[i]>ucl[i]|Zp[i]<lcl[i])

{rl[j]=i;break;}

else{rl[j]=0;}

}}

mean(rl)

sd(rl)
